# Rationale for combination of paclitaxel and CDK4/6 inhibitor in ovarian cancer therapy — non-mitotic mechanisms of paclitaxel

**DOI:** 10.3389/fonc.2022.907520

**Published:** 2022-09-15

**Authors:** Elizabeth R. Smith, Marilyn Huang, Matthew P. Schlumbrecht, Sophia H.L. George, Xiang-Xi Xu

**Affiliations:** ^1^ Sylvester Comprehensive Cancer Center, University of Miami Miller School of Medicine, Miami, FL, United States; ^2^ Department of Obstetrics, Gynecology and Reproductive Science, University of Miami Miller School of Medicine, Miami, FL, United States; ^3^ Department of Radiation Oncology, University of Miami Miller School of Medicine, Miami, FL, United States

**Keywords:** chemotherapy, taxanes/taxol/paclitaxel, microtubules, mitosis, nuclear envelope, micronuclei, CDK4/6, drug resistance

## Abstract

Taxanes and CDK4/6 inhibitors (CDK4/6i) are two families of successful anti-mitotic drugs used in the treatment of solid tumors. Paclitaxel, representing taxane compounds, has been used either alone or in combination with other agents (commonly carboplatin/cisplatin) in the treatment of many solid tumors including ovarian, breast, lung, prostate cancers, and Kaposi’s sarcoma. Paclitaxel has been routinely prescribed in cancer treatment since the 1990s, and its prominent role is unlikely to be replaced in the foreseeable future. Paclitaxel and other taxanes work by binding to and stabilizing microtubules, causing mitotic arrest, aberrant mitosis, and cell death. CDK4/6i (palbociclib, ribociclib, abemaciclib) are relatively new cell cycle inhibitors that have been found to be effective in breast cancer treatment, and are currently being developed in other solid tumors. CDK4/6i blocks cell cycle progression at the G1 phase, resulting in cell death by mechanisms not yet fully elucidated. At first glance, paclitaxel and CDK4/6i are unlikely synergistic agents as both are cell cycle inhibitors that work at different phases of the cell cycle, and few clinical trials have yet considered adding CDK4/6i to existing paclitaxel chemotherapy. However, recent findings suggest the importance of a non-mitotic mechanism of paclitaxel in cancer cell death and pre-clinical data support rationale for a strategic paclitaxel and CDK4/6i combination. In mouse tumor model studies, drug sequencing resulted in differential efficacy, indicating complex biological interactions of the two drugs. This article reviews the rationales of combining paclitaxel with CDK4/6i as a potential therapeutic option in recurrent ovarian cancer.

## Introduction

**Figure 1:**
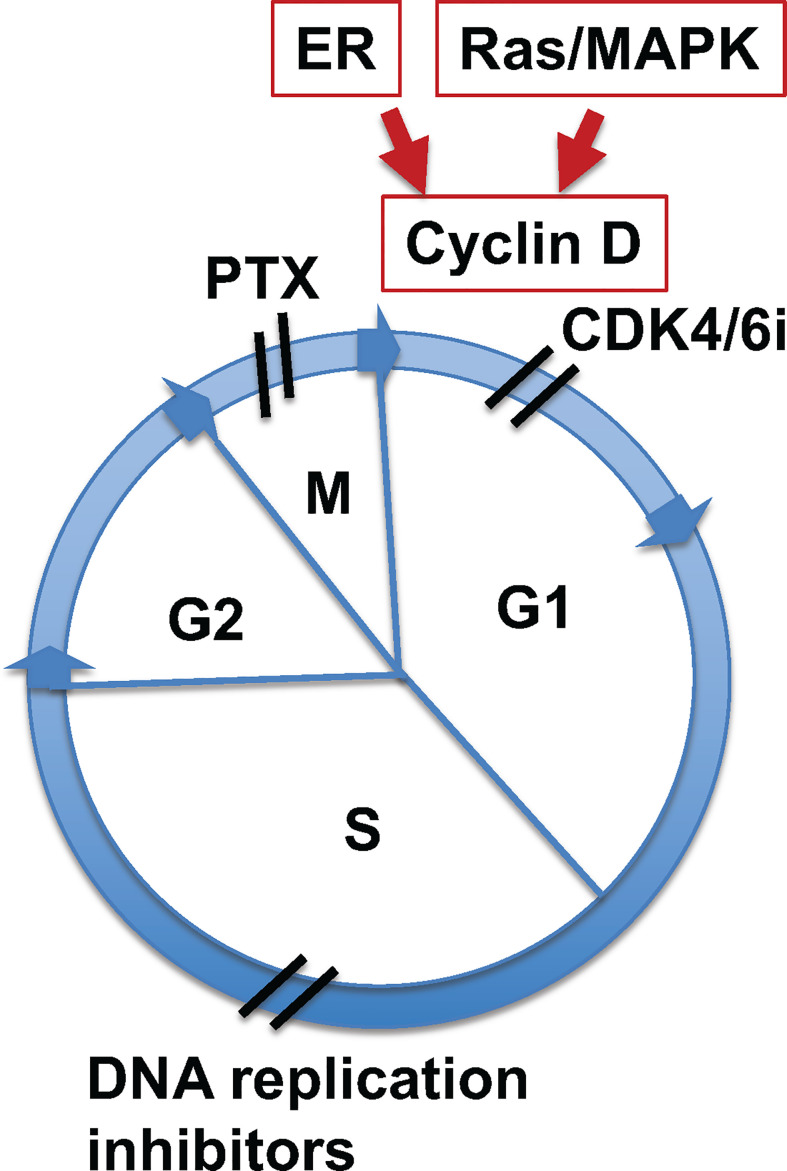
Paclitaxel and CDK4/6 inhibitors target different sites of the cell cycle. Illustration of sites of cell cycle targeted by paclitaxel and CDK4/6i. Mitogenic signaling by estrogen receptor (ER) or Ras/MAPK pathways induces cyclin D expression, which activates cyclin kinase 4 and 6 (CDK4/6) to initiate cell cycle through G1 phase. CDK4/6 inhibitors (CDK4/6i) block cycle kinase activities and arrest cells at early G1 phase. Paclitaxel (PTX) targets the function of spindle microtubules in cells at mitotic (M) phase, leading to aberrant mitosis and mitotic catastrophe. Additionally, mitotic inhibitors targeting DNA relication arrest cells at S phase.

Taxane compounds are effective anti-mitotic cancer drugs which have successfully been used for more than 30 years, and are often cornerstones in the management of ovarian cancer today. These drugs work as microtubule stabilizing agents, interfering with mitosis of proliferating cancer cells. Another family of newly developed anti-cancer drugs, the CDK4/6 inhibitors (CDK4/6i), are effective in breast cancer treatment, and these inhibitors are actively being tested and expanded in other malignancies. CDK4/6i block cancer cell growth at the G1 phase of the cell cycle, while paclitaxel (and additional taxanes) targets cancer cells at the M phase (**Figure 1**). Additional mitotic inhibitors can act by blocking DNA replication (**Figure 1**), but there are no such agents with tolerable toxicity and sufficient efficacy available to be commonly used in clinics.

Although agents in either families are effective anti-cancer drugs, issues on efficacy, response rate, and development of drug resistance are limiting factors for both. An obvious interest is to combine these two useful classes of common anti-cancer drugs for more effective cancer treatment. New biological understanding of these agents may provide a rationale and strategy to develop an enhanced cancer treatment regimen using them in combination to capitalize on their potential synergistic mechanisms of action.

## Taxanes as important common anti-cancer agents

Among many potential targets investigated for cancer therapy, stabilizing microtubules is one of the most effective strategies for cell kill *via* mitotic inhibition in many solid tumors (1–4). Paclitaxel is the first example of a microtubule stabilizing agent developed into a successful anti-cancer drug (4–7). Taxanes and non-taxane microtubule targeting agents remain common anti-cancer drugs, given their significant efficacy in multiple cancer types (4, 8–10). Taxol/paclitaxel, the first taxanes, was isolated from plant (Taxus brevifolia) as a cytotoxic anti-tumor agent (11–13). Currently, several taxane compounds, including paclitaxel, docetaxel, and cabazitaxel, are used as standard of care chemotherapeutic agents (14). Additional formulations of taxanes have been developed to improve delivery, including bound to albumin, and with additional nanoparticle carriers (15–18). Non-taxane microtubule stabilizing drugs, such as ixabepilone, are also tested and used in certain cancer types (9, 19, 20).

Paclitaxel is commonly used as a key component in front line therapy for epithelial ovarian cancer, and is given in combination with a platinum agent (cisplatin or carboplatin) (21–25). It also is utilized as a single agent in a dose dense (weekly) schedule to treat recurrent and drug (platinum agent)-resistant ovarian cancer (26–28). However, recurrent ovarian cancer progressively becomes refractory to continuous paclitaxel treatment, and the severity of side effects, such as peripheral neuropathy, correlates with accumulative drug dosage and often necessitates dose-reductions (29–33). Thus, strategies to enhance paclitaxel efficacy and to counter drug resistance are highly desirable and are actively sought (33–35). One strategy is to find potential synergistic combination with additional new agents, such as CDK4/6i.

## Paclitaxel in microtubule stabilization, mitotic mechanisms, and mitotic catastrophe

Paclitaxel, and all other taxane and non-taxane microtubule stabilizing drugs, act by binding to alpha-tubulin subunits within microtubules, resulting in stabilization of the filaments (36–39). The discovery of this unique cytotoxic mechanism occurred in the 1970s-80s (4, 7, 40, 41), when paclitaxel was first extracted from the bark of the Pacific Yew tree (4, 6, 11–13). By interfering with microtubules in mitosis, paclitaxel causes cell growth arrest at M-phase by cytoskeleton paralysis (Horwitz, 1994; 42), and subsequent cell death by apoptosis (43, 44). However, the molecular details on the initiation of apoptosis by paclitaxel have been elusive. Some studies suggest that paclitaxel-mediated cancer cell death is independent of caspase activation and does not follow a classic mechanism of apoptosis (45, 46). In laboratory study and comparision of a panel of tumor lines treated with paclitaxel in xenograft tumor models, neither degree of mitotic arrest nor apoptosis appeared to correlate with the anti-tumor effect of paclitaxel (47). Furthermore, paclitaxel anti-tumor activity is also independent of p53 mutational status of the tumors (47).

Paclitaxel-treated cancer cells arrested at M-phase often then undergo aberrant mitosis (known as mitotic slippage), resulting in the formation of multiple micronuclei and consequential death (mitotic catastrophe) (41, 48–51). Moreover, both laboratory and clinical observations led to the thinking that in addition to acting as a mitotic inhibitor, paclitaxel has cytotoxic activity against cancer cells with non-mitotic mechanisms (29, 52–57). Proposed non-mitotic paclitaxel mechanisms include paclitaxel-induced phosphorylation of apoptotic protein bcl-2 (58), disruption of microtubule-mediated cellular transport (52), physical breaking of nuclear envelope by rigid microtubule bundles (59), stimulating of inflammatory activity by paclitaxel-induced nuclear fragmentation (60), and anti-angiogenic activity by damaging endothelial cells (61–64).

The concept of a non-mitotic mechanism for paclitaxel action is re-enforced by the lack of efficacy of mitotic inhibitory drugs developed more specifically to target mitotic machineries (65, 66). A better understanding of the non-mitotic mechanism and the complex processes underlying cancer cell kill is crucial to design drug combinations with taxanes to optimize rates of response and overcome taxane drug resistance.

## Non-mitotic mechanisms of paclitaxel in inducing micronucleation and cell death by nuclear membrane rupture

Laboratory studies are fairly convincing that highly proliferative cells are sensitive targets for paclitaxel, as the drug preferentially kills proliferative cancer cells, which are more likely to be in M-phase (4, 7, 29, 41). Taxanes, however, also affect continuously growing non-cancer cell populations such as hair follicle matrix keratinocytes (67) and hemopoietic cells (32). Thus, the major side effects of paclitaxel include alopecia and neutropenia.

**Figure 2:**
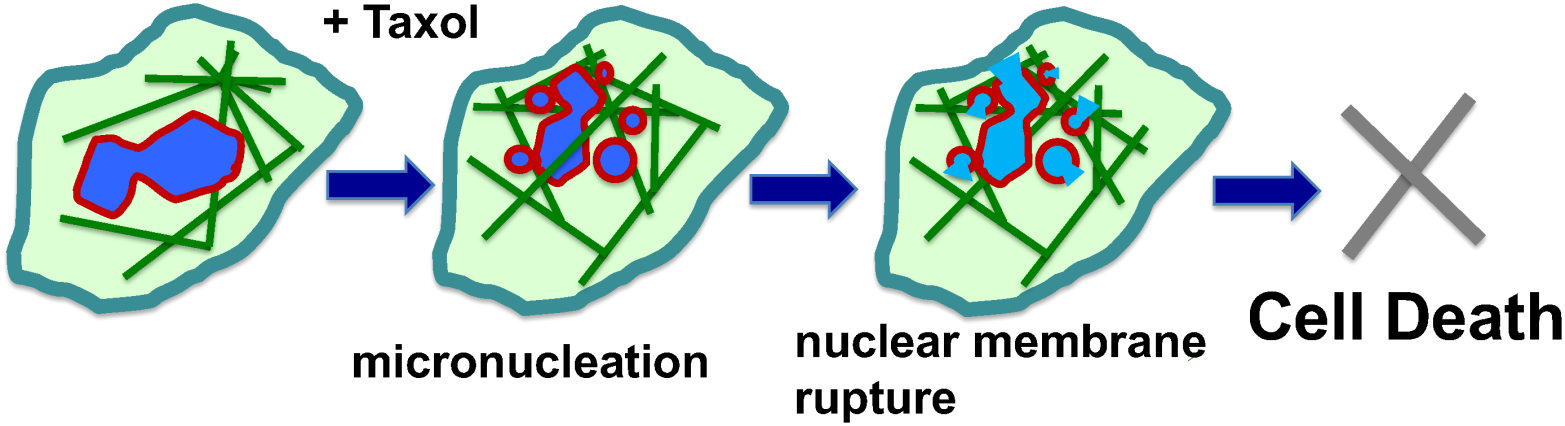
Mechanisms of paclitaxel-induced multiple micronucleation and nuclear membrane rupture in cancer killing. Paclitaxel (Taxol) induces mitotic catastrophe, resulting in micronucleation. In non-mitotic cells, the rigid microtubule filaments induced by paclitaxel can promote massive formation of micronuclei through nuclear budding of cells during interphase. The paclitaxel-bound rigid microtubule bundles pull and distort the nuclear envelope structure. As a result, the malleable cancer nuclear envelope breaks into multiple micronuclei (micronucleation). The proposal of physical force exerted by paclitaxel-induced rigid microtubule filaments in breaking malleable cancer nuclei provides a non-mitotic mechanism to generate multiple micronuclei. Paclitaxel also induces rigid microtubules and the breaking of nuclei of neoplastic cells and the formation of multiple micronuclei in both. The micronuclei derived from both mitotic and non-mitotic cells are defective in membrane structure and have high propensity for rupture and release of chromatin materials, resulting in cell death.

In contrast to cell culture models, only a small fraction of tumor cells *in vivo* are proliferative; despite this, most of the cancer cells in patient tumors are sensitive to paclitaxel (53, 54). Moreover, cell killing efficacy does not correlate with mitotic index (47, 54). Experimenal and clinical observations suggest that paclitaxel also kills cancer cells at non-mitotic phases, and interfering with the function of microtubules in G1 or S phases of the cell cycle also contributes to cancer cell killing (53–57). A new study suggests that in paclitaxel-treated cancer cells, the stabilized and rigid microtubule bundles around the cancer cell nucleus pull the nuclear envelope membrane by physical force into multiple micronuclei (59, 68) (**Figure 2**). This finding provides a new addition to the well-accepted notion that paclitaxel acts as a mitotic inhibitor. Thus, in addition to proliferation, a malleable nuclear envelope caused by a defective nuclear envelope structural proteins (69a) provides another specificity of cancer cells for killing by paclitaxel, as non-neoplastic cells have a sturdier nuclear envelope and are more resistant to paclitaxel-induced breaking (59, 68).

The formation of numerous micronuclei following paclitaxel treatment (45, 70), referred to as “micronucleation” (60), may be the consequence of both aberrant mitosis (41, 49–51, 71) and nuclear breaking in non-mitotic cells with a weakened nuclear envelope (59, 68). In the presence of several types of pharmaceutical compounds (including CDK4/6i) to inhibit mitosis, paclitaxel was observed to induce micronucleation, suggesting a non-mitotic mechanism to break up the cancer nucleus (59, 68). These small micronuclei are observed to be unstable and often undergo sudden and irreversible rupture (72, 73). A likely reason is that the nuclear membrane is stretched in micronucleation, as the combined surface of multiple smaller spheres is much larger than a single sphere with the same volume. Either by mitotic or non-mitotic mechanisms, the formation of multiple micronuclei is likely important for the efficacy of paclitaxel in killing cancer cells (60, 68). One possible mechanism is that the genomic DNA released will trigger the cGAS-Sting cytoplasmic DNA sensing pathway to activate the inflammatory pathway (60, 74). Nevertheless, the rupture of the nuclear membrane, essentially compromising a key cellular organelle, may be sufficient to assume the demise of the paclitaxel-treated cancer cells (46, 59) (**Figure 2**).

## Clinical efficacy of CDK4/6 inhibition

Small molecule compounds specifically targeting cell cycle kinases, including the CDK4/6 inhibitors, are new agents found to have activity in cancer treatment (75), and are commonly used in metastatic breast cancer. Additional indications in other solid tumors are currently under investigation (76–80).

The study of the mammalian cell cycle over several decades and the ultimate successful application of the knowledge to cancer therapy took a long road (80–82). Based on the identification and understanding of the cyclin-dependent kinase 4 (CDK4) and CDK6, the activator such as cyclin D1, and their multiple cyclin inhibitors, the concept of an inhibitor for cell cycle kinases to block cell cycle progression and tumor growth seems obvious (81). The first CDK4/6i to be developed and tested in clinical trial was palbociclib; however, the lack of efficacy of monotherapy in early studies limited the enthusiasm and delayed the clinical development. Fortunately, later trials showed a clear benefit of adding palbociclib to hormone antagonism therapy in metastatic breast cancer, leading to FDA approval of palbociclib in early 2015 (78–81). The details in the laboratory discoveries and clinical development of the CDK4/6i have been well reviewed in these (78–81) and many additional recent articles.

Following the initial success, many pharmaceutical companies independently developed additional CDK4/6i and are testing for their utility in combination therapy. Today, several CDK4/6 inhibitors, Ibrance (chemical name: palbociclib, developed by Pfizer.), Kisqali (chemical name: ribociclib, developed by Novartis), Verzenio (chemical name: abemaciclib, developed by Eli Lilly), have been developed and approved to treat metastatic breast cancer (78–80). Numerous clinical trials are ongoing to assess CDK4/6i in combination therapy to treat breast and additional cancer types. However, little information of CDK4/6i in ovarian cancer treatment has been reported yet, though substantial interests prompt ongoing efforts to evaluate a potential role in ovarian cancer treatment (83, 84).

De novo and acquired resistance to the combined treatments have been frequently observed, and alterations in both Rb and cell cycle regulation, and PI3K survival signaling pathway are potential mechanism of resistance (82, 83, 85). The CDK4/6i are exciting new drugs for cancer therapy, and ongoing studies and trials surely will add new mechanistic understanding to and improvement of clinical outcomes. Yet development of resistance to CDK4/6 inhibitors is already recognized as a limitation to this class. The rapidly accumulating information should allow the contemplation of strategy and design of rationale combinatorial therapies of CDK4/6i with other anti-cancer agents to overcome drug resistance and achieve superior treatment outcomes (80, 83, 86).

## Combination therapy: Rationale for synergy between paclitaxel and CDK4/6 inhibitors

The utility of CDK4/6 inhibition as a component in a combined therapy regimen with additional agent(s) is an area of active investigation as CDK4/6i by itself lacks sufficient activity (81, 84, 86). One potential mechanism of synergy is that both inhibition of the mitogenic signaling pathway that regulates D-type cyclins, and blocking of CDK4/6 activities, are necessary for a synergized therapy to prevent tumor cell proliferation (81). Paclitaxel and CDK4/6i are expected to be antagonists, since arresting cells by CDK4/6i at the G1 phase of the cell cycle presumably limits cell kill by paclitaxel, which targets cells at M-phase. Consistently, in laboratory studies, CDK4/6 inhibitors were shown to reduce and prevent apoptosis of hair follicle matrix cells that normally results from paclitaxel treatment (67), and the inhibitors also rescued hematopoietic cell death from paclitaxel treatment (87). Thus, CDK4/6i, when used strategically, may reduce some side effects of paclitaxel treatment.

Although not yet met with general enthusiasm because of the theory of antagonism and some preliminary observations, clinical trials for a paclitaxel/CDK4/6i have been attempted and initiated for solid tumors (for example, NCT 04594005). So far, no outcome has been reported. Pre-clinical studies of a paclitaxel/CDK4/6i combination have been attempted and reported, some with positive results (84, 88–90). In breast cancer cells, although simultaneous exposure to palbociclib and paclitaxel produced an antagonistic effect, sequential treatment caused higher cell death than single agent alone (88). The authors suggested pretreatment with CDK4/6i may enhance the efficacy of paclitaxel for chemotherapy of triple negative breast cancer (88). CDK4/6 inhibition was found synergistic in combination with paclitaxel to suppress growth and induce apoptosis in K-Ras mutant lung adenocarcinoma cells (90). In the cases of lung cancer cells, addition of paclitaxel first followed by CDK4/6i had higher cancer cell killing than the reversed sequence (89).

Another pre-clinical study found that the sequences for the administration of the two drugs produced differential efficacy in mouse pancreatic tumor xenograft models (91, 92). In the study, treatment first with paclitaxel followed by CDK4/6i produced better tumor suppressing activity than when CDK4/6i was administrated first. The authors suggest that CDK4/6i impairs the ability of cancer cells to recover from chromosomal and DNA damage caused by prior treatment with paclitaxel (91).

**Figure 3:**
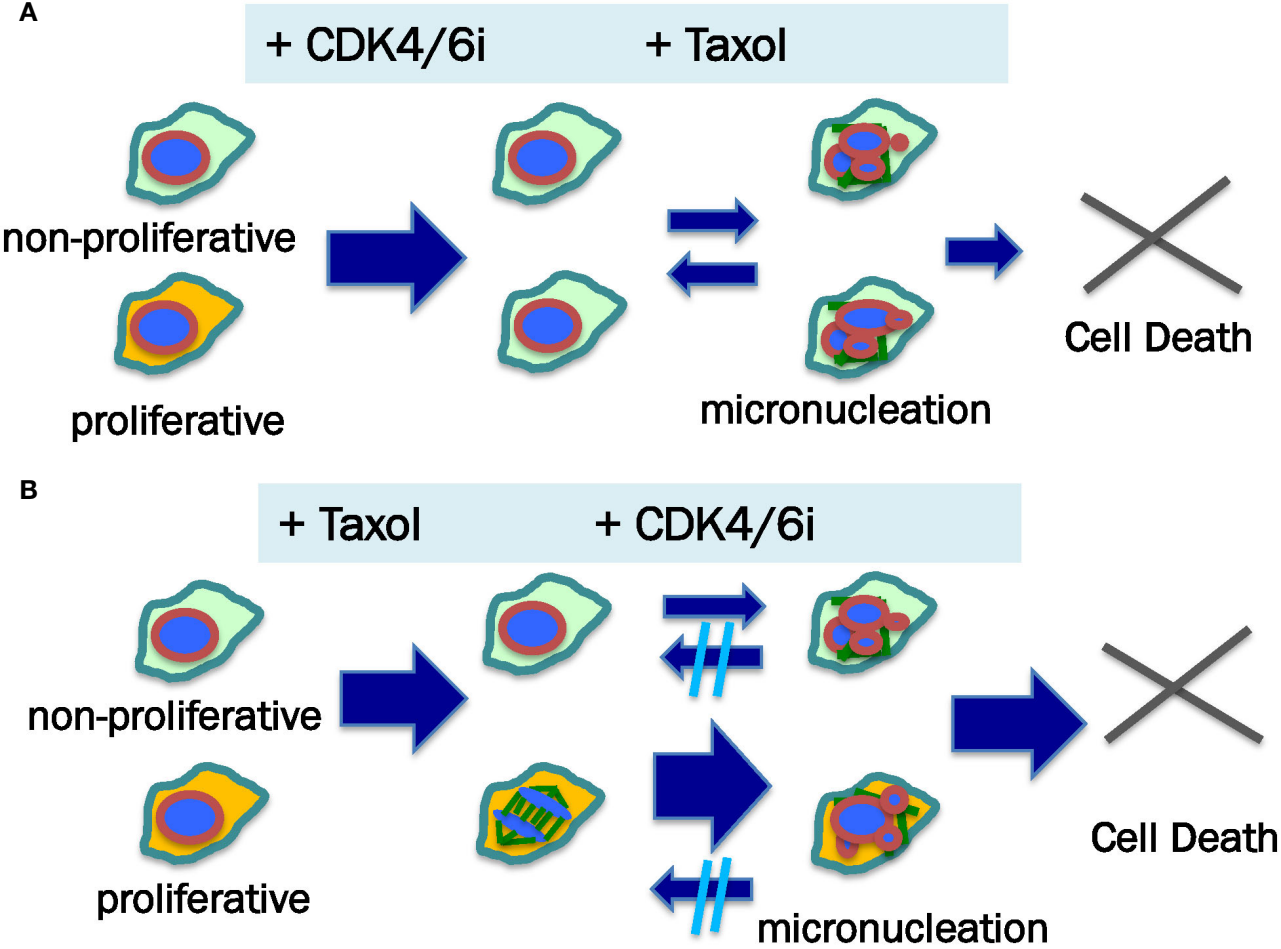
Proposed mechanism for sequence-dependent of paclitaxel and CDK4/6 inhibitor in killing cancer cells. Neoplastic cells within a tumor comprise proliferative (illustrated as yellow color cytoplasma) and non-mitotic (illustrated as green color cytoplasma) populations, which may respond to anti-cancer agents differently. **(A)** When CDK4/6i is added prior to paclitaxel (Taxol), the cancer cells are arrested at G1 phase, producing both cytotoxic and cytostatic effects. Subsequently, paclitaxel induces micronucleation and death of the non-mitotic cells. Some of the micronucleated cells may be able to recover. **(B)** In the case of paclitaxel addition first followed by CDK4/6i, both proliferative and non-mitotic cell populations undergo micronucleation, though mitotic cells more readily than non-mitotic cells form multiple micronuclei following paclitaxel stimulation (illustrated by small and bigger arrows). It is postulated that CDK4/6i treatment impairs the recovery of paclitaxel-induced damage to the nuclear structure (micronucleation). Thus, paclitaxel — CDK4/6i may have a higher cell killing outcome than CDK4/6i — paclitaxel sequences.

With the realization of the non-mitotic mechanism of paclitaxel in killing cancer cells (68), a new rationale may motivate the study of adding CDK4/6i to paclitaxel regimen, especially to the dose dense treatment of metastatic breast and recurrent ovarian cancer (**Figure 3**). Furthermore, ongoing study will yield additional understanding of the potential mechanism(s) of CDK4/6 inhibition in damaging cancer cells, in addition to the cytostatic effects. An initial treatment of cancer cells with CDK4/6 inhibitors may prevent mitosis-targeting mechanism of paclitaxel cytotoxicity (**Figure 3A**). A possible better strategy may be first to allow full attainment of the robust cytotoxic activity of paclitaxel alone to the mitotic cancer cell population before exposing the remaining cells to CDK4/6 inhibitors when non-mitotic paclitaxel killing mechanism still can occur (**Figure 3B**). Additionally, inhibition of CDK4/6 may impair the recovery of damaged and micronucleated cancer cells from prior exposure to paclitaxel, further enhancing the efficacy (91).

Paclitaxel exhibits high activity against mitotic cells, but also can kill non-mitotic cancer cells (59, 68), such as that are expected to accumulate in the presence of CDK4/6 inhibition. Thus, the possibility of a paclitaxel and CDK4/6i combination as a chemotherapy regimen for ovarian cancer exists. With a well-considered drug scheduling to avoid antagonism and fostering the synergy of the two drugs, a treatment with higher efficacy and overcoming drug resistance to both paclitaxel and CDK4/6i may be developed.

## Prospective: Clinical trial design — drug scheduling and sequence

Currently, paclitaxel and additional taxane compounds are the key drugs in the management of several major solid tumors, as frontline therapy and salvage option. Eventual development of resistance and accumulative side effects limit the continuous application of the drugs. Thus, the possibility of adding the new anti-cancer drug, CDK4/6i, to the paclitaxel regimen is highly desirable to increase drug potency and overcome resistance (80, 86). With the findings of a non-mitotic mechanism of paclitaxel (59, 68), and the observation of differential activity of drug administrative sequences (91), a therapeutic trial may be designed with these rationales, as an example discussed above (**Figure 3**).

**Figure 4:**
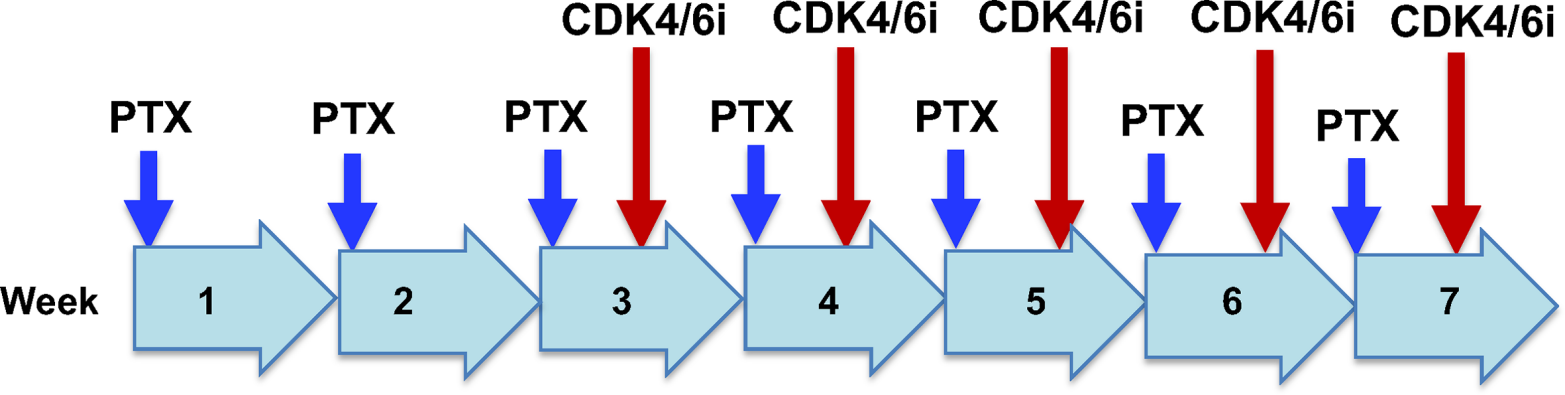
Potential clinical trial design for paclitaxel/CDK4/6i combination chemotherapy for recurrant ovarian cancer. Drug administration and schedule are illustrated for a hypothetical dose dense regimen of paclitaxel treatment of recurrant ovarian cancer. Paclitaxel (PTX) will be given alone in weeks 1 and 2. In weeks 3 to 7, paclitaxel will be given on day 1, and CDK4/6i will be administrated on day 6 of the week.It is postulated that paclitaxel alone in week 1 and 2 will eliminate the majority of proliferative cancer cells. In subsequent weeks, CDK4/6i given on day 6 will impair the recovery of damaged cancer cells from exposure to paclitaxel during the previous 5 days.

One approach may be the addition of CDK4/6i to dose dense paclitaxel treatment in patients with recurrent ovarian cancer and metastatic breast cancer (**Figure 4**). It may be suitable to use paclitaxel alone in the first two of a 7-cycle chemotherapy schedule, to eliminate most active proliferating cancer cells. In the subsequent 5 treatment cycles. CDK4/6i may be given in the last two days, based on the hypothesis that inhibition of CDK4/6 impairs the recovery of the damaged cancer cells following exposure for the previous 5 days with paclitaxel (91). Although paclitaxel is rapidly cleared from the circulation (1, 93), the drug is sequestered and persists within cells for several days (39, 94–96), where the drug stabilizes microtubules and produces additional cytotoxicity.

The proposed two drug combination and schedule has the benefit of both mitotic and non-mitotic mechanisms of paclitaxel action, plus growth inhibition and cytotoxicity bestowed by CDK4/6 inhibition, and thus are predicted to be a more effective therapy. It is not clear if there will be significant change in the side effect profile of either drugs when given in combination in the schedule designed. Both paclitaxel and CDK4/6i have tolerable side effects, and are both routinely used in clinics currently. The major side effects of paclitaxel are well documented: neutropenia/myelosuppression, alopecia, and peripheral neuropathy (32). No surprisingly, CDK4/6i suppresses cell proliferation and causes neutropenia/myelosuppression and alopecia (97). However, both agents inhibit cell cycle progression and may not be additive for cytotoxicity, and may be even antagonistic, as shown by preclinical findings for CDK4/6 inhibitors in protecting paclitaxel-caused hair follicle damage (67), or paclitaxel in myelosuppression (87). Thus, side effects such as myelosuppression/neutropenia and alopecia may be lessened or more severe, as results of either protection of paclitaxel damage by CDK4/6i, or the combined damage to the stem cells, respectively. This rationale for a potential sequential drug administration based on the paclitaxel dose dense regimen (**Figure 4**), derived from pre-clinical studies and consideration, will only be verified or disproved by a clinical trial in cancer patients.

## Author contributions

ES: developed concept, produced and analyzed research data, and edited manuscript; MH: developed concept and edited manuscript; MS: participated in concept development and edited manuscript; SG: participated in concept development and edited manuscript; X-XX: developed concept and wrote first draft of manuscript. All authors contributed to the article and approved the submitted version.

## Funding

Work described in this review article was partially supported by funds from NICHD R03HD071244 (ES), CDMRP DoD Concept Awards BC097189 and BC076832 (XX), and NCI grants R01 CA230916, R01 CA095071, R01 CA099471, and CA79716 (XX). Funds from the Sylvester Comprehensive Cancer Center/University of Miami also supported the research.

## Acknowledgments

Our lab alumni and students who have worked on this project and contributed to the basis of the current work. We also thank our colleagues for their conceptual discussion and advice during the course of the experiments and the preparation of the manuscript.

## Conflict of interest

MS is on the Stategic Council for GlascoSmithKlein (GSK).

The remaining authors declare that the research was conducted in the absence of any commercial or financial relationships that could be construed as a potential conflict of interest.

## Publisher’s note

All claims expressed in this article are solely those of the authors and do not necessarily represent those of their affiliated organizations, or those of the publisher, the editors and the reviewers. Any product that may be evaluated in this article, or claim that may be made by its manufacturer, is not guaranteed or endorsed by the publisher.
